# Effects of *Pistacia lentiscus* Leaf‐Twig and Resin (Mastic) Oils on Salivary Flow in Healthy Individuals

**DOI:** 10.1111/odi.70397

**Published:** 2026-07-06

**Authors:** Wiktoria Potocka, Luca van Eijndthoven, Marja L. Laine, Floris J. Bikker, Zainab Assy

**Affiliations:** ^1^ Department of Oral Biochemistry, Academic Centre for Dentistry Amsterdam University of Amsterdam and VU University Amsterdam Amsterdam LA the Netherlands; ^2^ Department of Periodontology, Academic Centre for Dentistry Amsterdam University of Amsterdam and VU University Amsterdam Amsterdam LA the Netherlands

**Keywords:** dry mouth, mastic, olfactory stimulus, *Pistacia lentiscus*, resin, saliva

## Abstract

**Objective:**

*Pistacia lentiscus* L. tree is known for its aromatic mastic gum containing terpenes α‐pinene and β‐myrcene, and has been applied as a traditional remedy in oral care for centuries. Previously, intranasal exposure to mastic resin volatiles increased salivary flow in healthy and dry‐mouth individuals. This study assessed the biological effects of oils from *P. lentiscus* for the purpose of standardisation and optimal performance.

**Methods:**

Composition analysis (GC–MS/FID) and acetylcholinesterase inhibition of *P. lentiscus* oils obtained from resin or leaf‐twig were assessed in vitro. Salivary properties (flow, spinnbarkeit, total protein concentration) and subjective mouthfeel were analysed before and after the application of the compounds in healthy volunteers (*n* = 12) in the form of nasal sprays.

**Results:**

This study confirmed that both oils tested contain compounds with acetylcholinesterase inhibitory activity in vitro. GC–MS/FID analysis showed different compositions between the resin and leaf‐twig oils. Application of leaf‐twig oil significantly increased salivary flow (median (IQR), 0.25 (0.15, 0.45) mL/min vs. 0.32 (0.19, 0.51) mL/min, *p* ≤ 0.01). There were no changes in spinnbarkeit, total protein concentration and mouthfeel.

**Conclusions:**

*P. lentiscus* leaf‐twig oil increased salivary flow in healthy participants. Further research is required to assess its effectiveness both in large cohort and in dry mouth patients.

## 1 Introduction

*Pistacia lentiscus* L. is an evergreen plant of the Anacardiaceae family, cultivated for its aromatic resin (gum) in the Mediterranean region, in particular Greece, Turkey, Morocco and Algeria (Kozhoridze et al. 2015). Mastic resin is rich in terpenes that give it its characteristic woody, resinous, green, pine‐like, fresh scent (Rigling et al. 2019). α‐Pinene, β‐myrcene, limonene, β‐linalool, and perillene are the key aroma compounds that are found in mastic resin harvested on the Greek island of Chios ( *P. lentiscus* L. var. *Chia*) (Kıvçak et al. 2004; Rigling et al. 2019).

The volatile oils are harvested from various plant organs, among them twigs, leaves, and flowers, each yielding a distinct volatile composition. For example, resin oil, which is harvested from the sap found in the tree stem, is rich in α‐pinene and contains considerably less β‐myrcene when compared to the oils extracted from leaves or twigs (Magiatis et al. 1999). Furthermore, the composition of oil can be affected by the plant's region of origin, including the climate and habitat (Barra et al. 2007; Said et al. 2011; Sehaki et al. 2022; Zrira et al. 2003), plant's sex (Said et al. 2011), being a cultivated or a wild tree (Tabanca et al. 2020), vegetative stage and harvest time (Barra et al. 2007; Zrira et al. 2003), and cultivar ( *P. lentiscus* L. versus *P. lentiscus* L. var. *Chia*) (Kıvçak et al. 2004).

The medical application of mastic resin can be traced back to ancient times (Pachi et al. 2020). The first ever mention of mastic was most likely in “Histories” by Greek historian and geographer Herodotus in the 5th century BCE (Herodotus 2024). However, mastic resin was used even before that in Egypt, where it was detected in the embalming fluid of an Egyptian mummy from the 7th century BCE (Colombini et al. 2000). Medical researchers of antiquity, such as Dioscorides (Dioscorides 2000) and Galen (Everett 2012) mentioned various applications of mastic in their writing (Pachi et al. 2020). Among others, Dioscorides (Dioscorides 2000) recommended use of mastic gum and oil for gastrointestinal disorders and as a cleaning agent for the oral cavity. Nonetheless, mastic is still being used to this day, applied as a traditional herbal remedy (European Medicines Agency and Committee on Herbal Medicinal Products (HMPC) 2015), and also in the oral health care (Alwadi et al. 2023).

Currently, applications of mastic resin are being studied for oral care purposes, including the management of periodontal disease,oral cancer, and oral dryness, as well as dental caries prevention (Alwadi et al. 2023; Faruque et al. 2024). Novel treatments and delivery methods are needed, as the percentage of the population affected by dry mouth is growing (Agostini et al. 2018), mainly due to the side effects of medication and polypharmacy (Carpenter 2015). Yet, patients find that the products currently available on the market are not fast‐acting, and do not provide sufficient relief for dry mouth (Assy et al. 2023, 2022).

It was recently shown that nasal exposure to mastic resin volatiles significantly increases salivary flow in both healthy individuals and dry mouth patients (Faruque et al. 2023, 2024). However, for practical reasons and standardisation purposes, such as shelf‐life and ease of daily use, a more suitable delivery method is preferred. Hence, a nasal spray was selected as an alternative delivery method (Potocka et al. 2025).

The aim of this study was to assess the sialagogic properties of resin and leaf‐twig *P. lentiscus* oil (resin, leaf‐twig) in the form of nasal sprays acting as an olfactory stimulus in healthy individuals. Saliva quantity and composition were assessed before and after use of the compounds. Furthermore, in vitro acetylcholinesterase (AChE) inhibitory activity and volatile composition of the *P. lentiscus* oils were assessed.

## 2 Methods and Materials

### 2.1 Plant Material

Oils of *P. lentiscus* from different plant organs (leaf‐twig, resin) were used in this study (Table 1).

**TABLE 1:** Information regarding compounds selected for an in vivo screening.

Plant name	Manufacturer	Plant organ	Origin	Extraction method	CAS‐No/WFO‐ID	Batch number
*Pistacia lentiscus* L. var. *Chia* oil	Anemos	Resin	Chios, Greece	Steam distillation	263‐098‐6/ wfo‐0000394118	ND
*Pistacia lentiscus* L. oil	NaturActive	Leaves and twigs	North Africa	Steam distillation	ND/ wfo‐0000394118	2K4PK

Abbreviation: ND, not determined.

### 2.2 GC–MS/FID

A total of 10 μL of essential oil was accurately weighed into a 2 mL GC vial (Agilent Technologies, Santa Clara, California, USA). A total of 1 mL of ethanol (HiPerSolv CHROMANORM grade, VWR Chemicals, West Chester, Pennsylvania, USA) was added, and 10 μL of a 1% standard of methyl decanoate (Sigma‐Aldrich, St. Louis, Missouri, USA) was transferred via pipette (Rainin LTS, Oakland, California, USA), thoroughly mixed and analysed by GC–MS/FID (Agilent Technologies).

Analysis by gas chromatography was conducted using an Agilent Technologies 8890 gas chromatograph, fitted with a purged three‐way microfluidic splitter plate (Agilent Technologies G3183B) after the primary column. The effluent was split one‐to‐one between a flame ionisation detector and an Agilent Technologies 5977B single quadrupole mass spectrometer with electron ionisation.

The column configuration comprised a SolGel‐Wax (Trajan, Ringwood, Australia) 30 m × 0.25 mm × 0.25 μm primary column. Helium (BOC, Guildford, UK) was used as the carrier gas, and the primary column flow rate was kept constant at 1 mL/min. The restrictor from the splitter to the flame ionisation detector was 1.1 m × 0.18 mm deactivated fused silica (Agilent Technologies) with a constant pressure of 3.8 PSI, and the restrictor from the splitter plate to the mass spectrometer was 2.7 m × 0.18 mm deactivated fused silica (Agilent Technologies) with a constant pressure of 3.8PSI to obtain a 1:1 split ratio. The split/splitless column inlet injector was heated to 250°C and operated in split mode (20:1 ratio) with 1 μL of sample introduced via a 10 μL syringe (Trajan) using a Centri PAL3 RTC (Markes International, Bridgend, UK). The gas chromatograph was held at 50°C (3 min), then programmed to 250°C at 5°C/min, held for 5 min. The flame ionisation detector was held constant at 275°C, the make‐up gas was purified nitrogen with a flow rate of 25 mL/min, with a purified air flow rate of 400 mL/min and 50 mL/min of hydrogen (BOC). Data were collected at 50 Hz. The mass spectrometer transfer line was held at 280°C, the ion source and quadrupole temperatures were held at 230°C and 150°C, respectively. The mass scan range was m/z 35–450 at an acquisition rate of 3.5 Hz.

Data was acquired in Agilent MassHunter Workstation and analysed using a combination of Agilent MassHunter Unknowns Analysis, AMDIS (Automated Mass Spectral Deconvolution and Identification System for GC/MS), and MassHunter Qualitative Analysis software packages using commercially available NIST23, Wiley12 and in‐house libraries along with retention indices (calculated by injection of an internal reference) to identify components. Percent by relative peak area calculated using primarily flame ionisation response, and where necessary, deconvoluted total ion count (TIC) response to allocate the proportion of coeluted components within the flame ionisation response.

The GC–MS/FID analysis of the oils was carried out by Givaudan UK.

### 2.3 Inhibitory Acetylcholinesterase Activity In Vitro

AChE inhibition of *P. lentiscus* oils was assessed using Acetylcholinesterase Inhibitor Screening Kit (MAK324, Sigma‐Aldrich, Merck, Missouri, USA) according to the manufacturer's instructions, using acetylcholinesterase from *Electrophorus electricus* (electric eel) (C3389, Sigma‐Aldrich, Merck, Missouri, USA) and colorimetric 96‐well plates (655191, Greiner Bio‐One, Kremsmünster, Austria). European Pharmacopoeia Reference Standard donepezil hydrochloride 0.1 mg/μL (Y0002160, EDQM, Strasbourg, France) was used as an additional standard. The final concentration of samples was 2.5% v/v. The reading was carried out using Multiskan FC Microplate Photometer (Thermo Fisher Scientific, Massachusetts, USA) at absorbance 405 nm at 0 min and at 10 min. All measurements were conducted in duplicate and repeated at least once.

### 2.4 Sample Preparation

Samples were prepared as described previously (Potocka et al. 2025). Plastic PET transparent nasal 30 mL spray pumps (Your Body My Concern, Raalte, the Netherlands) were used to deliver compounds. Samples were prepared by first mixing by hand one of the *P. lentiscus* oils (resin or leaf‐twig) with a 1:10 ratio of Tween 20 (Polysorbate) EMPROVE Essential Ph. Eur., JPE, NF (Merck, Darmstadt, Germany). Then, 0.9% NaCl solution was slowly added for a final volume of 10 mL. 0.9% NaCl solution was prepared using sodium chloride EMPROVE API Ph. Eur., BP, JP, USP (Merck, Darmstadt, Germany) and Mili‐Q water (Merck Milipore, Massachusetts, USA). Final concentrations of all compounds were 0.01%. Each spray bottle contained 10 mL of solution. The placebo spray consisted of 10 mL of 0.9% NaCl solution. Tween 20 was not added to the placebo spray as in the absence of dissolved oil compounds, it may produce a noticeable ‘soapy’ sensation associated with nasal discomfort, potentially affecting participant perception and comfort. Both sample and placebo bottles were sprayed at least 10 times before application to remove air from the pump system and ensure equal dosing. The estimated volume of each full dose (two sprays) corresponded to approximately 22 nL of resin oil and 26 nL of leaf–twig oil per administration. Assuming a typical essential oil density of approximately 0.85–0.95 g/mL, this corresponds to an estimated mass in the microgram (μg) range per dose. These values are approximate, as the exact density of the oils was not experimentally determined.

The 0.01% concentration was selected as a conservative starting dose based on earlier feasibility work with terpene‐containing olfactory stimuli and to minimise irritation risk during pilot testing.

### 2.5 Participants

Twelve healthy adult volunteers from Amsterdam, the Netherlands, were recruited for this pilot study, which took place between October and December 2024. All procedures were performed in compliance with relevant laws and institutional guidelines and have been approved by the local Ethical Committee of the Academic Centre for Dentistry Amsterdam with protocol no: 2022‐43674. The participants received information regarding the study both verbally and written and then gave their written informed consent and filled in a demographic information form and a health questionnaire. Only healthy subjects above the age of 18 years old were included. Exclusion criteria included: prescription medication, disorders that could affect their salivary flow, current smokers, problems with olfactory perception, current flu, allergies to any of the compounds and/or products in which these compounds could be found, rhinitis, hay feverand/or eczema, and pregnancy or lactation.

For 1 h before the experiment, the participants were instructed not to eat or drink and not to brush their teeth or use any oral care products. Furthermore, participants were asked not to wear any perfumes or other odorous products on the day of the saliva collection. During the saliva collection, participants were asked to sit straight in a resting state, not swallow the saliva, and not move or talk.

### 2.6 Questionnaires

Visual analogue scale (VAS) questions were set up on a 0–10 scale with responses recorded to one decimal point. Participants were asked to answer one VAS question before saliva collection: “How moist does your mouth feel at this moment?” with a scale of response from “very dry” (0) to “very moist” (10). After the experiment, participants were asked to answer two VAS questions: “How moist does your mouth feel at this moment?” with a scale of response from “very dry” (0) to “very moist” (10) and “How pleasant did you find the odour you just smelled?” with a scale of response from “very unpleasant” (0) to “very pleasant” (10).

### 2.7 In Vivo Exposure and Saliva Samples Collection

Saliva sampling was carried out in the morning (9:00–10:30 AM) to avoid natural variations caused by the circadian rhythm. Each participant received both compounds, each on different day (2 days total). Participants received the compounds in a randomised order and were not informed in advance which compounds they were receiving (single‐blind design). For example, participant A received *P. lentiscus* leaf‐twig oil on day 1 and resin oil on day 2. The study days were separated by at least 24 h, which was considered sufficient to minimise potential carryover effects, as previous work showed that the effects of mastic resin volatiles diminished within approximately 20 min after stimulation (Faruque et al. 2023). To minimise expectation bias, the nasal inhalers were prepared at a different location and kept in sealed containers until the collection of stimulated whole saliva (SWS).

Both unstimulated whole saliva (UWS) and SWS were collected by allowing saliva to accumulate at the bottom of the mouth, then drooling it into a tube after 5 min. Additionally, for SWS collection, participants were asked to administer the *P. lentiscus* oil/placebo solution by spraying one full pump press into each nostril (two sprays total) while inhaling at the beginning of the saliva collection. Saliva collections were carried out according to the protocol (Figure **1**). At day 1, participants received information both written and verbally, were asked to sign the consent form, and fill in a health and “Before the experiment” questionnaire. Then, UWS—“before” was collected, followed by a 3 min break. Afterwards, placebo‐SWS was collected. This was followed by a 3 min break, and then nasal spray (resin or leaf‐twig)—“after” SWS was collected. At the end, participants filled in the questionnaire “After the experiment”. During day 2, “Before the experiment” questionnaires were filled out, then UWS—“before” was collected, followed by a 3 min break. Afterwards, SWS using nasal spray (resin or leaf‐twig)—“after”, was collected. At the end, participants filled in the questionnaire “After the experiment”.

**FIGURE 1:**
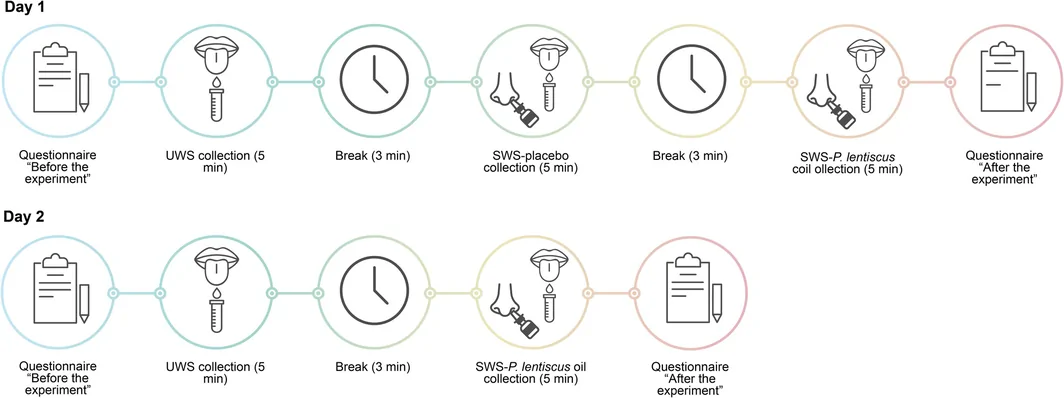
Schematic protocol of the olfactory stimulation measurements in volunteers. SWS, stimulated whole saliva; UWS, unstimulated whole saliva.

### 2.8 Spinnbarkeit Measurements

After the collection, saliva samples were kept on ice, and shortly before the spinnbarkeit measurements, vortexed for 3 s. Salivary spinnbarkeit was measured using the Neva meter (Ishikawa Iron Works Corporation, Tokyo, Japan, (Gohara et al. 2004)) according to a previously published protocol (Potocka et al. 2025). The device was set to “dry mode”, measure speed: 0.50 mm/s, dip depth: 0.95 mm, hold time up: 1 s, hold time down: 10 s and the detection method: optical. Measurements were performed in quintuplicates using 80 μL of saliva. The average value (in mm) was calculated by excluding the highest and lowest readings and averaging the remaining three.

### 2.9 Total Protein Concentration

Total protein content was measured using Pierce Bicinchoninic acid assay (BCA) Protein Assay Kit (Thermo Fisher Scientific, Massachusetts, USA) and 96‐well polystyrene microplates (Greiner Bio‐One, Frickenhausen, Germany), according to the manufacturer's protocol. Bovine serum albumin was used as a standard. The absorbance was measured at 562 nm using the Multiskan FC Microplate Photometer. The total protein concentration was calculated according to the manufacturer's protocol. The assay was carried out in replicates.

### 2.10 Statistical Analysis

The statistical analysis was carried out using GraphPad Prism (Dotmatics, Massachusetts, USA). Normality of data was assessed using the Shapiro–Wilk test (*p* < 0.05). Values are reported as mean ± SD (normally distributed data), unless stated otherwise. Values of *p* < 0.05 were considered statistically significant.

Statistical analysis was carried out between “before” and “after” measurements to account for interpersonal differences between participants and any fluctuations occurring from measurements on different days. Differences in subjective mouth feeling, pleasantness of scents and total protein concentration were assessed by paired *t*‐test (normally distributed data). Differences in salivary flow and spinnbarkeit were assessed by a paired Wilcoxon test (not normally distributed data). Correlation between salivary flow and pleasantness of the compounds was calculated using Spearman's rank correlation coefficient. Post hoc effect size was estimated using G*Power 3.1.9.7 (Heinrich‐Heine‐Universität Düsseldorf, Düsseldorf, Germany), selecting the Wilcoxon signed‐rank test (matched pairs), parent distribution “min ARE” (not normally distributed data) or “normal” (normally distributed data), two tails, α err prob. 0.05, and total sample size *n* = 12.

## 3 Results

### 3.1 Volatile Composition and Inhibitory Acetylcholinesterase Activity In Vitro

The main compounds of resin oil were identified as (flame ionisation detector percentage (%FID) by relative peak area (RPA)) α‐pinene (82.89%), β‐myrcene (8.38%) and β‐pinene (2.12%) (Table 2). For leaf‐twig oil, α‐pinene was the main compound (21.32%), followed by β‐myrcene (17.65%) and D‐limonene (15.91%) (Table 2). Full results of the analysis can be found in Tables S1 and S2. At the concentration of 2.5% v/v, both oils of *P. lentiscus* (resin, leaf‐twig) were found to nearly completely inhibit AChE under the assay conditions.

**TABLE 2:** Volatile composition of the *Pistacia lentiscus* resin (mastic) and leaf‐twig oils.

Compound name	CAS#	FID % by RPA
*Pistacia lentiscus* resin (mastic) oil		
α‐pinene	80‐56‐8	82.89
β‐myrcene	123‐35‐3	8.38
β‐pinene	18172‐67‐3	2.12
α‐pinene oxide	1686‐14‐2	0.92
Trans‐verbenol	1820‐09‐3	0.71
Cis‐verbenol	1845‐30‐3	0.7
D‐limonene	5989‐27‐5	0.62
Camphene	79‐92‐5	0.54
Perillene	539‐52‐6	0.4
2‐methylanisole	578‐58‐5	0.36
*Pistacia lentiscus* leaf‐twig oil		
α‐pinene	80‐56‐8	21.32
β‐myrcene	123‐35‐3	17.65
D‐limonene	5989‐27‐5	15.91
Terpinen‐4‐ol	562‐74‐3	6.72
P‐cymene	99‐87‐6	5.74
Caryophyllene	87‐44‐5	4.29
α‐terpineol	98‐55‐5	3.53
β‐pinene	18172‐67‐3	3.02
Camphene	79‐92‐5	2.22
β‐phellandrene	555–10‐2	2.13

Abbreviations: %FID, flame ionisation detector percentage; RPA, relative peak area.

### 3.2 In Vivo Pilot Study Cohort

A total of 12 healthy volunteers (30.17 ± 8.94 years, range 19–45 years) took part in this study, including 6 females (29.17 ± 8.80 years, range 20–44 years) and 6 males (31.17 ± 9.81 years, range 19–45 years).

### 3.3 Salivary Flow and Extensional Rheology (Spinnbarkeit)

Ortho‐nasal stimulation with the compounds resulted in a significant increase in salivary flow rates after the application of leaf‐twig oil (median (IQR), UWS 0.25 (0.15, 0.45) mL/min vs. SWS 0.32 (0.19, 0.51) mL/min, effect size 0.67, *p* ≤ 0.01) (Figure 2). The salivary flow did not change after stimulation with the resin oil (UWS 0.26 (0.12, 0.41) mL/min vs. SWS 0.26 (0.19, 0.54) mL/min). There was no observed placebo effect (UWS 0.28 (0.14, 0.39) mL/min vs. SWS 0.30 (0.17, 0.43) mL/min).

**FIGURE 2:**
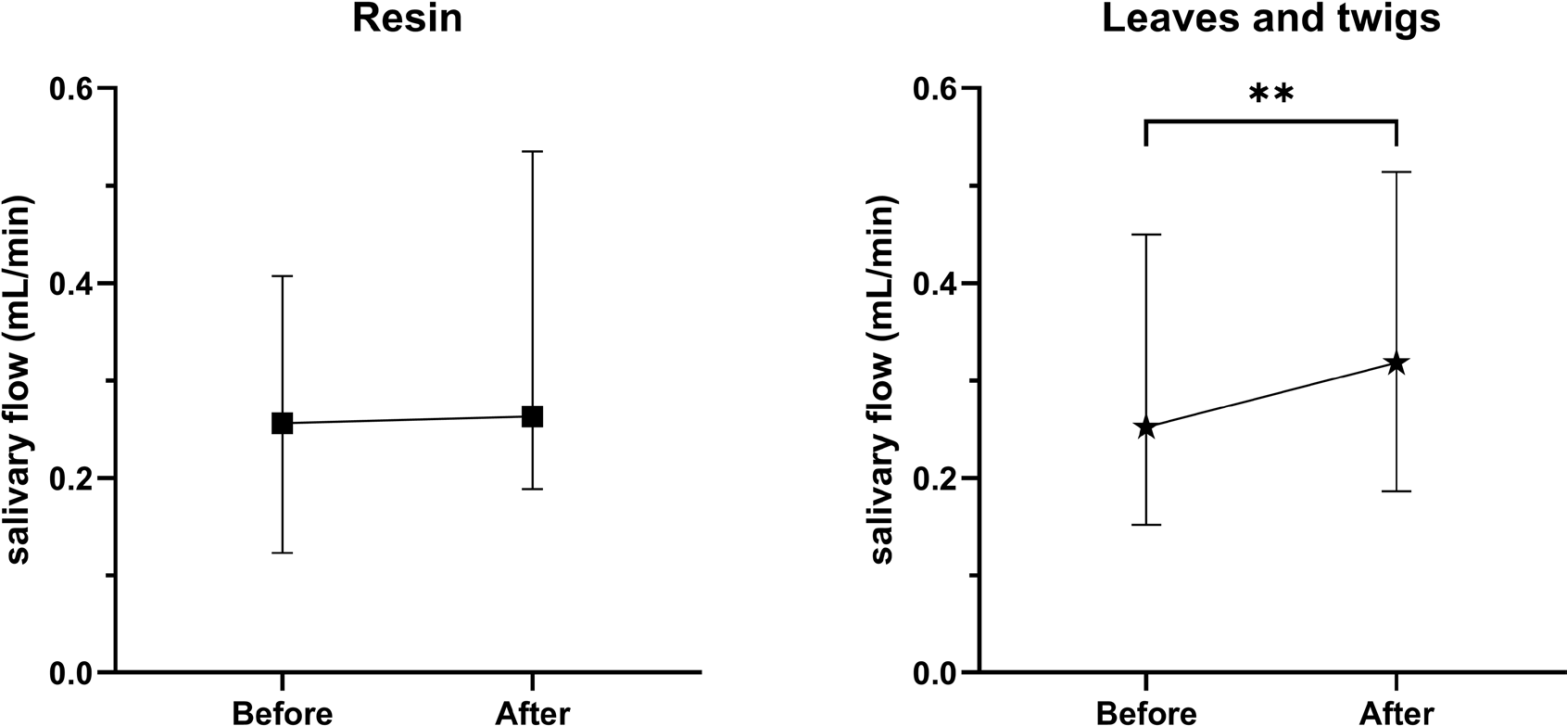
Salivary flow (mL/min) before (UWS—unstimulated whole saliva) and after stimulation with *P. lentiscus* oils: resin and leaf‐twig (SWS—stimulated whole saliva) by nasal spray in healthy participants (*n* = 12). Values are expressed as median ± IQR. Paired Wilcoxon test. **p ≤ 0.01.

Spinnbarkeit did not change before and after application of *P. lentiscus* leaf‐twig oil (UWS 17.00 (6.82, 40.26) mm vs. SWS 24.26 (4.15, 57.78) mm), and resin oil (UWS 14.01 (5.71, 52.67) mm vs. SWS 15.38 (4.86, 77.20) mm).

### 3.4 Total Protein Concentration

The analysis of total salivary protein concentration revealed no difference between before and after the application of resin (UWS 939.52 ± 323.98 μg/mL vs. SWS 921.11 ± 398.72 μg/mL) and leaf‐twig (UWS 929.70 ± 302.86 μg/mL vs. SWS 906.19 ± 309.74 μg/mL).

### 3.5 Mouth Feeling and Pleasantness of the Scents

There was no difference in mouth feeling before and after the application of the *P. lentiscus* leaf‐twig oil (UWS 4.67 ± 1.24 vs. SWS 4.54 ± 1.24) and the resin oil (UWS 4.78 ± 1.22 vs. SWS 4.63 ± 1.63). Scent of resin oil (6.32 ± 1.86) was found to be significantly more pleasant than the scent of leaf‐twig oil (4.13 ± 1.92) (effect size 1.12, *p* ≤ 0.01) (Figure 3). There was no correlation between salivary flow and pleasantness of the compounds (resin oil *r* = −0.17, leaf‐twig oil r = 0.57).

**FIGURE 3:**
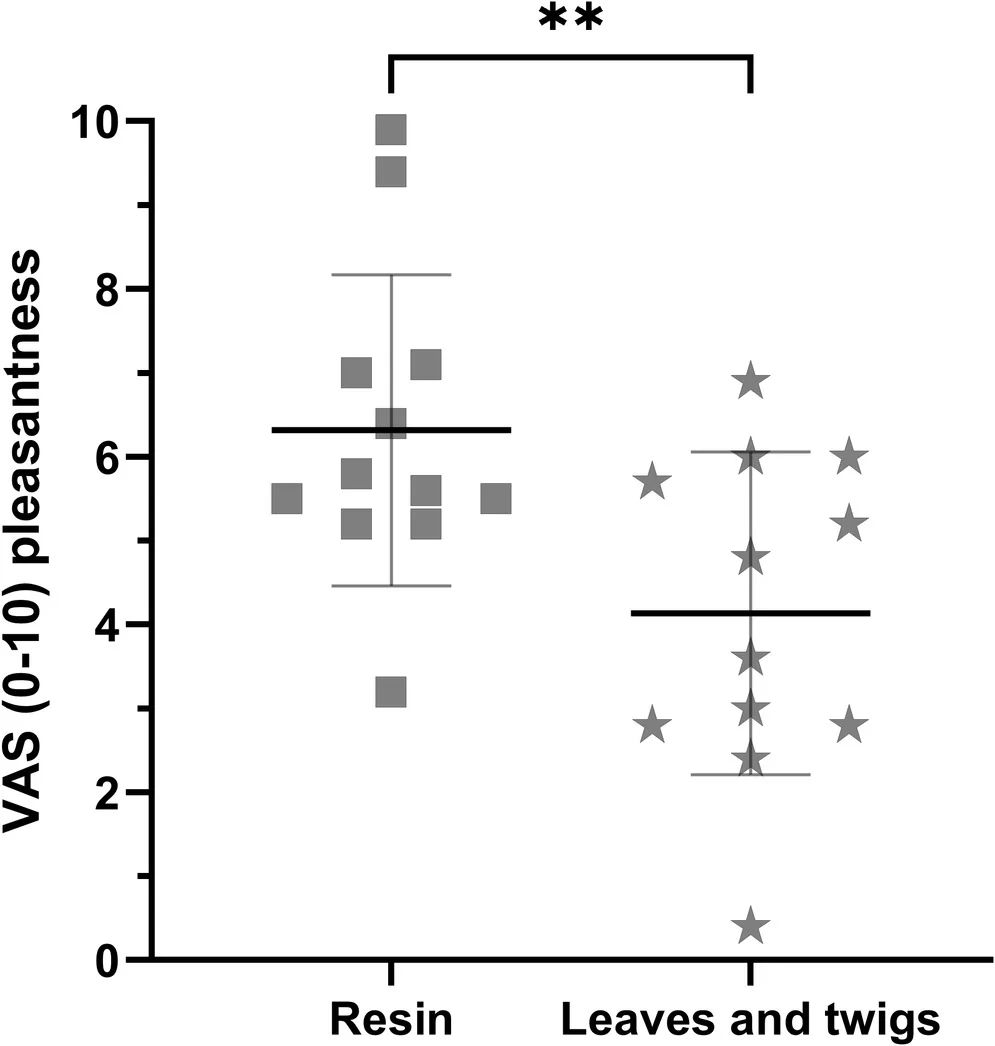
Score of the pleasantness after the stimulation with nasal spray with *P. lentiscus* resin and leaf‐twig oils in healthy participants (*n* = 12). The feeling was scored using VAS from 0 (“very unpleasant”) to 10 (“very pleasant”) in response to a question “How pleasant did you find the odour you just smelled?”. Values are expressed as mean ± SD. Paired *t*‐test. **p ≤ 0.01.

## 4 Discussion

This study assessed the sialagogic properties of *P. lentiscus* oils obtained from different plant organs: resin, which is secreted by the stem of the plant, or from young, green plant parts such as leaves and twigs. For standardisation and handling purposes, the oils were diluted and dispersed in the nasal cavity using nasal sprays. The exposure to the leaf‐twig oil increased the salivary flow in vivo, in contrast to the resin‐derived oil. No differences in spinnbarkeit and subjective perception were observed after the application of oils. Furthermore, resin oil was found to be significantly more pleasant than the leaf‐twig oil. Pleasantness did not have an influence on the salivary flow outcomes.

Salivary output is modulated by a combination of regulatory pathways. Primarily, saliva secretion is regulated by the autonomic nervous system. During parasympathetic autonomic nerve stimulation, AChE inhibitors decrease the breakdown of AChE, resulting in an increase or prolongation of parasympathetic neurotransmission and subsequent stimulation of salivary flow (Proctor and Carpenter 2007). Secretion is also regulated via transient receptor potential (TRP) channels in response to different stimuli (Liu et al. 2018) and acinar cell secretion via Ca^2+^‐ dependent mechanisms (Ambudkar 2014).

To better understand the potential mode of action of the active compounds, an analysis of the composition was carried out. The composition of the oils analysed was consistent with the previous literature reports on specimens collected in the same regions (Pachi et al. 2021; Ramdani et al. 2016). α‐Pinene was found to be the main compound of both oils harvested from resin and from green plant parts (leaves and twigs). However, resin oil contained around four times more α‐pinene than leaf‐twig oil. α‐Pinene is a weak AChE inhibitor (Dohi et al. 2009; Miyazawa and Yamafuji 2005), and its ability to increase salivary flow is thought to occur through AChE inhibition (Dohi et al. 2009; Miyazawa and Yamafuji 2005).

The second most abundant compound, β‐myrcene, has weak AChE inhibitory properties (Burčul et al. 2020). Leaf‐twig oil contained about twice as much β‐myrcene as the resin oil. β‐myrcene is thought to be modulating TRP vanilloid 1 (TRPV1) channels. It has to be noted that the data proving this ability are conflicting (Heblinski et al. 2020; Jansen et al. 2019). TRPV1 receptors are heat‐activated ion channels that contribute to the transmission and modulation of nociceptive signals (e.g., capsaicin) (Rosenbaum and Simon 2007). In addition to the sensory role, TRPV1 channels have a function in store‐operated Ca^2+^ entry (SOCE) mechanism, which is involved in regulating salivary fluid secretion (Ambudkar 2014; Liu et al. 2018). In recent studies, TRPV1 agonists such as capsaicin (Gardner et al. 2020; Pushpass et al. 2019) or nonivamide (Houghton et al. 2020) have been shown to increase salivary flow in humans.

D‐limonene, which can be found abundantly in citrus peels, is the third most abundant monoterpene in the leaf‐twig oil. Limonene is not a strong AChE inhibitor (Burčul et al. 2020), nor is it thought to be able to bind to TRPV1 channels (Kaimoto et al. 2016). It was demonstrated to inhibit transient receptor potential ankyrin 1 (TRPA1) channel (Kaimoto et al. 2016), but the role of TRPA1 channels in salivation is not well understood (Houghton et al. 2020; Liu et al. 2018). In patients with radiation‐induced xerostomia, D‐limonene improved patient outcomes by activating aldehyde dehydrogenase 3A1 (ALDH3A1), which preserves salivary gland structure and function following the radiation treatment (Saiki et al. 2018; Wright et al. 2025).

The minor constituents of the oils might also be playing a role in the salivary stimulation. Minor constituents with low odour thresholds may disproportionately influence olfactory‐driven autonomic responses. Although the composition analysis in this study primarily focused on monoterpenes, plant essential oils contain a range of other bioactive constituents, including polyphenols (e.g., flavonoids), terpenes (e.g., sesquiterpenes) and terpenoids (e.g., oxygenated monoterpenes) (Arzani et al. 2025).

Importantly, the concentration used in vitro (2.5% v/v) was substantially higher than the estimated exposure levels achieved via nasal administration (0.01% formulation, with nanolitre quantities per dose), indicating that direct AChE inhibition is unlikely to fully explain the observed in vivo effect. Moreover, both oils demonstrated similar AChE inhibition in vitro, whereas only the leaf–twig oil increased salivary flow in vivo, further suggesting that additional mechanisms are involved. The sialagogic effect of *P. lentiscus* leaf–twig oil may be attributed to the synergistic effects of its diverse terpenic constituents with muscarinic and TRP receptors.

These findings are in parallel with the results of Faruque et al. (2024), who demonstrated a positive effect of mastic resin volatiles delivered in the form of natural resin on the salivary flow. However, in contrast to the current study, no effect was observed after the use of the mastic resin oil. This difference may be explained by two key factors: the composition of the resin oil and the delivery method. First, the chemical composition of *P. lentiscus* resin oil can vary significantly depending on its geographic origin. In this study, the resin oil was sourced from *P. lentiscus* trees from the island of Chios in Greece, whereas Faruque et al. used resin from *Pistacia* trees growing in Iran (resin composition not determined). Second, differences in the method of delivery may also have played a role. Faruque et al. used volatiles from freeze‐dried crushed resin in sachets, while in this study, the oils were administered as a 0.01% solution using nasal sprays. This methodological difference could influence the distribution and concentration of the active molecules binding to the receptors in the oronasal cavity, and consequently, their final effect on the salivary flow.

A primary limitation of this study is the relatively small sample size, which is attributable to its pilot‐study design. Besides, the observed effects of the compounds are limited to short‐term outcomes, and long‐term effects need to be evaluated. The findings of this study apply to outcomes in a population of healthy volunteers, and further research is required to determine the effects in dry‐mouth patients. Furthermore, the compositional data obtained from GC–MS/FID analysis are presented as relative peak area percentages, which provide semi‐quantitative information, as the values are relative proportions, not absolute concentrations. This may be influenced by compound‐specific response factors. In addition, the analysis was carried out on a single batch of each oil, which does not take into consideration batch‐to‐batch variation. Future studies require tolerability testing and dose escalation to appropriately evaluate concentrations of the compounds.

Next steps in this research should include re‐assessing the scent profiles and composition of the compound solutions, dosage optimisation, determination of the long‐term effects of the compounds and an assessment in a larger healthy cohort, and then further in a dry mouth patient cohort.

## 5 Conclusions

In conclusion, this study showed the potential of *P. lentiscus* leaf‐twig oil as a sialagogue in healthy participants. Yet further research is required to improve the scent profile and delivery method of the treatment and determine the long‐term effects in both healthy and dry‐mouth cohorts.

## Author Contributions

**Luca van Eijndthoven:** validation, methodology, formal analysis, investigation, writing – review and editing. **Marja L. Laine:** conceptualization, validation, resources, writing – review and editing, supervision, funding acquisition, project administration. **Zainab Assy:** conceptualization, validation, methodology, formal analysis, investigation, writing – review and editing, supervision, project administration. **Floris J. Bikker:** conceptualization, validation, resources, writing – review and editing, supervision, project administration, funding acquisition. **Wiktoria Potocka:** conceptualization, methodology, validation, formal analysis, investigation, data curation, writing – original draft, visualization, project administration, writing – review and editing.

## Funding

This research was funded by the Nederlandse Organisatie voor Wetenschappelijk Onderzoek (NWO) grant number 19217.

## Conflicts of Interest

The authors declare no conflicts of interest.

## Supporting information


**Table S1:** Volatile composition of the *Pistacia lentiscus* resin (mastic) oil. %FID—flame ionisation detector percentage, RPA—relative peak area, RT—retention time.
**Table S2:** Volatile composition of the *Pistacia lentiscus* leaf‐twig oil. %FID—flame ionisation detector percentage, RPA—relative peak area, RT—retention time.

## Data Availability

The data that support the findings of this study are not openly available due to reasons of sensitivity and are available from the corresponding author upon reasonable request. https://doi.org/10.48338/VU01‐ST8I43
