# Supplementary material for: Effects of Pistacia lentiscus Leaf‐Twig and Resin (Mastic) Oils on Salivary Flow in Healthy Individuals
Source: Oral Dis. 2026 Jul 6;32(10):3180–8. doi: 10.1111/odi.70397 (PMC13768939; doi:10.1111/odi.70397)
Supplement: Supplementary file 1 [file ODI-32-3180-s001.docx]

**Supplementary data**

**Table S1.** Volatile composition of the *Pistacia lentiscus* resin (mastic) oil.
%FID – flame ionization detector percentage, RPA – relative peak area, RT – retention time

| RT (min) | Compound name | CAS# | FID % by RPA |
| --- | --- | --- | --- |
| 6.05 | Alpha-pinene | 80-56-8 | 82.89 |
| 9.26 | Beta-myrcene | 123-35-3 | 8.38 |
| 7.79 | Beta-pinene | 18172-67-3 | 2.12 |
| 14.74 | Alpha-pinene oxide | 1686-14-2 | 0.92 |
| 22.1 | Trans-verbenol | 1820-09-3 | 0.71 |
| 19.83 | Cis-verbenol | 1845-30-3 | 0.7 |
| 10.1 | D-limonene | 5989-27-5 | 0.62 |
| 6.84 | Camphene | 79-92-5 | 0.54 |
| 15.98 | Perillene | 539-52-6 | 0.4 |
| 15.73 | 2-methylanisole | 578-58-5 | 0.36 |
| 21.61 | Trans-pinocarveol | 547-61-5 | 0.36 |
| 19.07 | Linalool | 78-70-6 | 0.32 |
| 19.6 | Pinocarvone | 30460-92-5 | 0.32 |
| 28.57 | Caryophyllene oxide | 1139-30-6 | 0.3 |
| 22.74 | Verbenone | 80-57-9 | 0.25 |
| 8.12 | Sabinene | 3387-41-5 | 0.21 |
| 24.62 | (-)-Myrtenol | 19894-97-4 | 0.14 |
| 21.01 | Myrtenal | 564-94-3 | 0.11 |
| 15.51 | 2,6-Octadiene, 2,6-dimethyl- | 2792-39-4 | 0.09 |
| 18.05 | Cis-p-methan-1(7),8-dien-2-ol | 22626-43-3 | 0.07 |
| 12.04 | P-cymene | 99-87-6 | 0.06 |
| 16.48 | Cis-linaloloxide | 1000121-97-4 | 0.03 |
| 17.54 | Ylangene | 14912-44-8 | 0.03 |
| 10.3 | Eucalyptol | 470-82-6 | 0.02 |
| 10.35 | Beta-phellandrene | 555-10-2 | 0.02 |
| 19.35 | Linalyl acetate | 115-95-7 | 0.02 |
| 21.94 | Furfural | 98-01-1 | 0.02 |

**Table S2.** Volatile composition of the *Pistacia lentiscus* leaf-twig oil.
%FID – flame ionization detector percentage, RPA – relative peak area, RT – retention time

| RT (min) | Compound name | CAS# | FID % by RPA |
| --- | --- | --- | --- |
| 6 | Alpha-pinene | 80-56-8 | 21.32 |
| 9.28 | Beta-myrcene | 123-35-3 | 17.65 |
| 10.16 | D-limonene | 5989-27-5 | 15.91 |
| 20.4 | Terpinen-4-ol | 562-74-3 | 6.72 |
| 12.06 | P-cymene | 99-87-6 | 5.74 |
| 20.34 | Caryophyllene | 87-44-5 | 4.29 |
| 22.54 | Alpha-terpineol | 98-55-5 | 3.53 |
| 7.79 | Beta pinene | 18172-67-3 | 3.02 |
| 6.85 | Camphene | 79-92-5 | 2.22 |
| 10.37 | Beta-phellandrene | 555-10-2 | 2.13 |
| 11.37 | Gamma-terpinene | 99-85-4 | 2.03 |
| 23.95 | Delta cadinene | 483-76-1 | 1.83 |
| 12.37 | Terpinolene | 586-62-9 | 1.21 |
| 19.91 | (+)-Borneol acetate | 20347-65-3 | 0.96 |
| 28.57 | Caryophyllene oxide | 1139-30-6 | 0.8 |
| 22.42 | Gamma-muurolene | 30021-74-0 | 0.75 |
| 9.62 | Alpha-terpinene | 99-86-5 | 0.7 |
| 21.99 | Humulene | 6753-98-6 | 0.7 |
| 23.21 | Alpha-muurolene | 10208-80-7 | 0.61 |
| 15.15 | Methyl heptyl ketone | 821-55-6 | 0.43 |
| 32.77 | M-camphorene | 20016-73-3 | 0.41 |
| 11.93 | Isoamyl butyrate | 106-27-4 | 0.4 |
| 5.71 | Tricyclene | 508-32-7 | 0.4 |
| 22.88 | Germacrene D | 23986-74-5 | 0.35 |
| 11.56 | Beta-ocimene | 13877-91-3 | 0.32 |
| 33.18 | Alpha-cadinol | 481-34-5 | 0.3 |
| 9.25 | Beta-pinene | 127-91-3 | 0.27 |
| 30.52 | Trans-p-Menth-2-ene-1,4-diol | 21473-37-0 | 0.24 |
| 8.13 | Sabinene | 3387-41-5 | 0.24 |
| 20.16 | Beta-elemene | 515-13-9 | 0.22 |
| 28.38 | Beta-caryophyllene oxide | 1139-30-6 | 0.21 |
| 32.38 | Tau-muurolol | 19912-62-0 | 0.2 |
| 16.95 | Iso amyl octanoate | 2035-99-6 | 0.19 |
| 32.45 | Para-menth-2-en-1,6-diol (tent | 146073-89-7 | 0.19 |
| 21.43 | Aromadendrene | 489-39-4 | 0.18 |
| 32.01 | Cis-3-p-Menthen-1,2-diol | 33669-76-0 | 0.18 |
| 19.07 | Linalool | 78-70-6 | 0.17 |
| 17.77 | Copaene | 3856-25-5 | 0.15 |
| 30.03 | Epicubenol | 19912-67-5 | 0.15 |
| 12.71 | Iso amyl iso valerate | 659-70-1 | 0.15 |
| 29.66 | Humulene oxide II | 19888-34-7 | 0.15 |
| 25.56 | Calamenene | 73209-42-4 | 0.14 |
| 32.1 | Tau-cadinol | 5937-11-1 | 0.14 |
| 33.62 | P-camphorene | 20016-72-2 | 0.13 |
| 9.54 | 1,4-cineole | 470-67-7 | 0.13 |
| 30.14 | Di-epi-1,10-cubenol | 73365-77-2 | 0.12 |
| 25.82 | P-cymene-8-ol | 1197-01-9 | 0.12 |
| 23.75 | Alpha-farnesene | 502-61-4 | 0.12 |
| 16.41 | P-(1-Propenyl)-toluene | 1000429-54-9 | 0.11 |
| 18.35 | 1-nonanol | 143-08-8 | 0.1 |
| 32.59 | Torreyol | 19435-97-3 | 0.09 |
| 23.03 | Delta-guaiene | 3691-11-0 | 0.09 |
| 17.09 | 2,2,6-trimethyl-1-oxaspiro[2,5]oct-5-ene | 2886-59-1 | 0.09 |
| 11.11 | Trans-beta-ocimene | 3779-61-1 | 0.09 |
| 27.2 | Isoamyl benzoate | 94-46-2 | 0.08 |
| 31.67 | Cis-p-menth-2-ene-1,4-diol | 22626-43-3 | 0.08 |
| 22.64 | Borneol | 507-70-0 | 0.08 |
| 21.77 | Cadina-1(6),4-diene,trans- | 20085-11-4 | 0.07 |
| 7.87 | Limetol | 7392-19-0 | 0.07 |
| 24.95 | Sabinyl acetate | 3536-54-7 | 0.07 |
| 24.48 | Cubenene | 29837-12-5 | 0.06 |
| 29.21 | Unknown | n/a | 0.05 |
| 6.07 | Alpha-phellandrene | 99-83-2 | 0.05 |
| 14.74 | Alpha-pinene oxide | 1686-14-2 | 0.05 |
| 8.81 | Delta-3-carene | 13466-78-9 | 0.05 |
| 22.13 | Unknown | n/a | 0.03 |
| 17.24 | Delta-elemene | 20307-84-0 | 0.03 |
| 12.25 | Iso-amyl 2-methyl butyrate | 27625-35-0 | 0.03 |
| 13.33 | Pinol | 2437-97-0 | 0.02 |
| 27.28 | Alpha-calacorene | 21391-99-1 | 0.02 |
| 16.58 | Trans-limonene oxide | 4959-35-7 | 0.02 |
| 10.31 | Eucalyptol | 470-82-6 | Trace |
| 27.35 | Caryophyllene oxide | 1139-30-6 | Trace |
| 13.29 | Cis-3 hexenyl acetate | 3681-71-8 | Trace |
| 17.31 | Methyl-2-butyrate hexenyle-3-cis | 53398-85-9 | Trace |
| 13.04 | (E)-4,8-dimethylnona-1,3,7-triene | 19945-61-0 | Trace |
